# The Preparation of g-C_3_N_4_/ZnIn_2_S_4_ Nano-Heterojunctions and Their Enhanced Efficient Photocatalytic Hydrogen Production

**DOI:** 10.3390/molecules29112571

**Published:** 2024-05-30

**Authors:** Hubing Li, Yaoting Wang, Song Wang, Xin Xiao

**Affiliations:** 1 Jiangsu Key Laboratory of Marine Bioresources and Environment, Jiangsu Ocean University, 59 Cangwu Road, Haizhou, Lianyungang 222005, China; 2 Co-Innovation Center of Jiangsu Marine Bio-Industry Technology, Jiangsu Ocean University, 59 Cangwu Road, Haizhou, Lianyungang 222005, China; 3 Jiangsu Institute of Marine Resources Development, 59 Cangwu Road, Haizhou, Lianyungang 222005, China; 4 Jiangsu Key Laboratory of Function Control Technology for Advanced Materials, Jiangsu Ocean University, 59 Cangwu Road, Haizhou, Lianyungang 222005, China

**Keywords:** g-C_3_N_4_/ZnIn_2_S_4_, semiconductor, photocatalytic hydrogen production

## Abstract

Hydrogen production technology has triggered a research boom in order to alleviate the problems of environmental pollution and the pressure on non-renewable energy sources. The key factor of this technology is the use of an efficient photocatalyst. g-C_3_N_4_ is a typical semiconductor photocatalytic material that is non-toxic and environmentally friendly and does not cause any serious harm to human beings. Therefore, it can be applied to drug degradation and the photocatalytic production of H_2_. Combined with ZnIn_2_S_4_, this semiconductor photocatalytic material, with a typical lamellar structure, has become one of the most promising catalysts for research due to its suitable bandgap structure and excellent photoelectric properties. In this study, 10% g-C_3_N_4_/ZnIn_2_S_4_ nano-heterojunction composite photocatalytic materials were successfully prepared by compounding ZnIn_2_S_4_ and g-C_3_N_4_ semiconductor materials with good visible-light-trapping ability. Under visible light irradiation, the photocatalytic activity of the composites was significantly better than that of pure g-C_3_N_4_ and ZnIn_2_S_4_. This is attributed to the formation of a heterojunction structure, which effectively inhibited the recombination of photogenerated carriers through the interfacial contact between the two semiconducting materials, and then improved the separation efficiency of the photogenerated electron–hole pairs, thus enhancing the catalytic activity. In this study, pure g-C_3_N_4_ and ZnIn_2_S_4_ were prepared using calcination and hydrothermal methods, and then, the composites were synthesized using ultrasonic and hydrothermal means. The differences in the structure, morphology, and hydrogen production performance of the materials before and after recombination were analyzed in detail using XRD, SEM, and FTIR characterization, which further verified that the 10% g-C_3_N_4_/ZnIn_2_S_4_ nano-heterojunction composites possessed excellent photocatalytic activity and stability, providing new possibilities for the optimization and application of photocatalytic hydrogen production technology.

## 1. Introduction

In recent years, due to the sharp increase in the world’s energy consumption, the deterioration of the ecological environment is showing an increasingly serious trend, especially regarding non-renewable resources, such as oil, natural gas, and coal, which are difficult to restore after their consumption and which cause a series of environmental problems [1,2]. The most troubling aspects for humans are global climate change caused by the emissions of greenhouse gases and acid rain caused by the emissions of sulfur-containing and nitrogen-containing compounds, which have extremely negative impacts. Therefore, the development of new energy sources is urgent. Hydrogen, a clean secondary energy source, has attracted a lot of attention. In recent years, more and more attention has been paid to the development, research, and utilization of solar energy, and many researchers and scholars have begun to decompose water by using sunlight to obtain hydrogen [3,4]. The biggest advantage of this method is maximizing the use of energy. Research has found that water can be split in the presence of a semiconductor catalyst using sunlight irradiation. The semiconductor is excited by an external force to produce photogenerated electrons and holes, which react with the water to produce hydrogen. Photocatalytic technology is crucial for solving the energy crisis, and the development of highly efficient and stable photocatalysts is key to this technology [5,6].

g-C_3_N_4_ is a typical semiconducting polymer with a two-dimensional lamellar structure similar to graphene’s planar structure. g-C_3_N_4_ is considered one of the most promising semiconducting materials using visible light. It has a high value for the degradation of pollutants and the preparation of H_2_ and O_2_ through photocatalysis [7,8]. The photocatalytic properties of C_3_N_4_ materials can be improved by increasing their surface area and modulating their electronic structure. Porous 3D or exfoliated 2D C_3_N_4_-based materials have been successfully used in the preparation of efficient photocatalysts with a high surface area and an improved visible light absorption ability [9]. g-C_3_N_4_ has attracted attention in many fields because it can be prepared in a very simple way, and it has no toxic substances of its own that can cause serious damage to the human body. Additionally, it is a friendly material that does not cause serious pollution to the environment, and it can be used as an economical precursor at a low price and in the development of a wide range of applications [10,11,12]. As a new type of non-metallic photocatalytic material, it has been shown that g-C_3_N_4_ has a relatively wider absorption spectral range than traditional TiO_2_ photocatalysts, and, therefore, its photocatalytic reaction is more stable than that of traditional TiO_2_ [13]. Compared to traditional TiO_2_ photocatalysts, its photocatalytic reaction can take place only under normal visible light. g-C_3_N_4_ also has a more effective ability to activate molecular oxygen compared to TiO_2_, and it is able to generate photocatalytic conversion reactions for organic functional groups and superoxide radicals to meet the requirements for the photocatalytic degradation of organic pollutants [14,15,16]. However, pure g-C_3_N_4_ has a small forbidden bandwidth (2.01 eV), is very easily excited by visible light, and suffers from the disadvantage of a fast photogenerated electron–hole complexation rate, in addition to its relatively slow electron transfer rate.

ZnIn_2_S_4_ is a photocatalyst material belonging to an AB_2_X_4_ (A = Zn, Ca, Cd, and Cu; B = Al, In, and Ga; X = Se, S, and Te) semiconductor family system. With a typical layered structure, ZnIn_2_S_4_ is generally considered to of the most promising catalysts based on its excellent optical and electrical properties, suitable bandgap (2.34–2.48 eV), high visible light utilization, etc. ZnIn_2_S_4_ has both cubic and hexagonal crystal system structures [17,18,19,20]. Many experts and scholars have reported that ZnIn_2_S_4_ semiconductor photocatalytic materials with these two crystal systems are widely used for the photocatalytic preparation of H_2_ and the degradation of environmental pollutants under the irradiation of visible light due to their very high catalytic activity and good chemical stability. ZnIn_2_S_4_ also has a suitable bandgap structure, which makes it possible to build a binary system catalyst or a multisystem catalyst with other semiconductors [1,21,22]. This can largely improve the separation efficiency of the photogenerated carriers and thus increase their migration rate, which can also improve the photocatalytic performance. In order to improve the catalytic activity of optical semiconductor photocatalysts, two catalysts with different conduction and valence bands are usually compounded into a new type of heterostructure catalyst so that the photogenerated electron–hole complex can be better suppressed and the catalytic activity can be efficiently enhanced. With its good absorbance, the composite of ZnIn_2_S_4_ and g-C_3_N_4_ can effectively promote the utilization of photogenerated carriers in the system, and the performance of the composite photocatalytic material can be improved.

At present, we are facing a variety of environmental and energy problems. An effective way to solve the problem of energy consumption is the semiconductor photocatalytic decomposition of water to produce hydrogen. Therefore, it is crucial to find a good catalyst, as the key factor of photocatalytic hydrogen production technology is a highly efficient photocatalyst. It has been found through research that composite semiconductor materials can improve photocatalytic activity to a large extent, making this an effective method.

In the present work, pure g-C_3_N_4_ nanoflakes were prepared through calcination, and then, pure ZnIn_2_S_4_ was prepared using the hydrothermal method to study their respective photocatalytic properties. Then, 10% g-C_3_N_4_/ZnIn_2_S_4_ nano-heterojunction composites were prepared by loading g-C_3_N_4_ nanoflakes onto ZnIn_2_S_4_ using the hydrothermal method. A series of characterization tests were carried out to analyze the morphologies and differences in the hydrogen production properties of the materials before and after composition in order to obtain better photocatalytic materials.

## 2. Results and Discussion

### 2.1. X-ray Diffraction (XRD) Analysis

**Figure 1:**
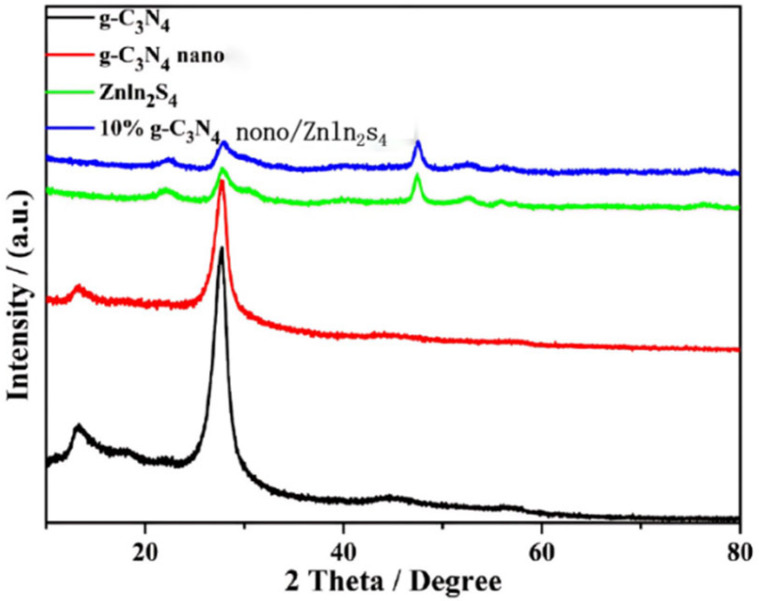
XRD patterns of g-C_3_N_4_, nano g-C_3_N_4_, ZnIn_2_S_4_, and 10% nano g-C_3_N_4_/ZnIn_2_S_4_.

Figure 1 shows the XRD patterns of the different samples, from which we can see that there are diffraction peaks at 2θ = 21.5° and 2θ = 27.6° for the photocatalytic materials that we prepared. The characteristic peaks at 2θ = 27.6° correspond to the crystalline surface (002) of g-C_3_N_4_, and the characteristic diffraction peaks at 2θ = 27.39°, 47.60°, and 56.69° for the prepared ZnIn_2_S_4_ samples can be found in the XRD pattern of the material prepared [23,24]. The XRD patterns of the prepared ZnIn_2_S_4_ samples show characteristic diffraction peaks at 2θ = 27.39°, 47.60°, and 56.69°, and the cell structure of ZnIn_2_S_4_ is hexagonal according to the cell parameters. The images show that the addition of g-C_3_N_4_ did not affect the structure of ZnIn_2_S_4_. The XRD patterns of the 10% g-C_3_N_4_/ZnIn_2_S_4_ samples are similar to those of ZnIn_2_S_4_, which indicates that we successfully prepared 10% g-C_3_N_4_/ZnIn_2_S_4_ nano-heterojunction composite photocatalytic materials and that the g-C_3_N_4_ was homogeneously loaded onto the ZnIn_2_S_4_ photocatalytic material [25,26]_._

### 2.2. Scanning Electron Microscopy (SEM) and Transmission Electron Microscopy (TEM) Analyses

**Figure 2:**
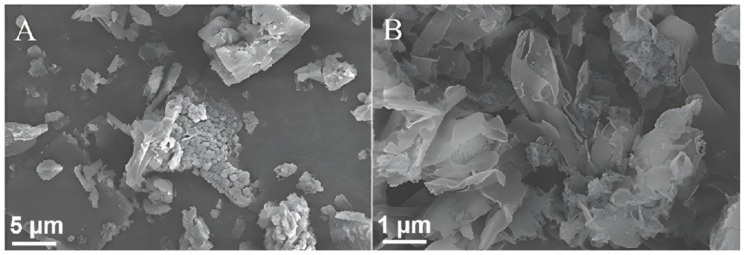
(**A**) SEM image of g-C_3_N_4_ and (**B**) SEM image of nano g-C_3_N_4_.

**Figure 3:**
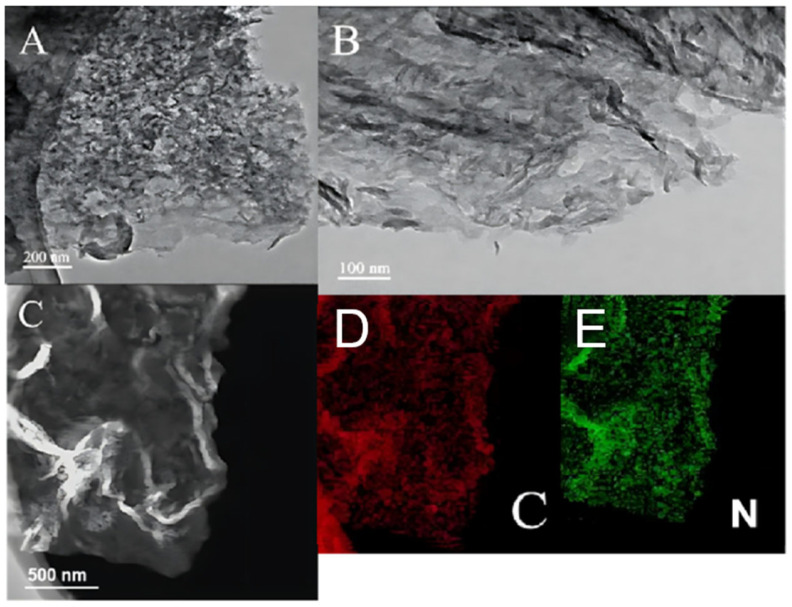
(**A**) TEM image of g-C_3_N_4_, (**B**) TEM image of nano g-C_3_N_4_, (**C**) dark-field image, and (**D**,**E**) mapping of nano g-C_3_N_4_.

**Figure 4:**
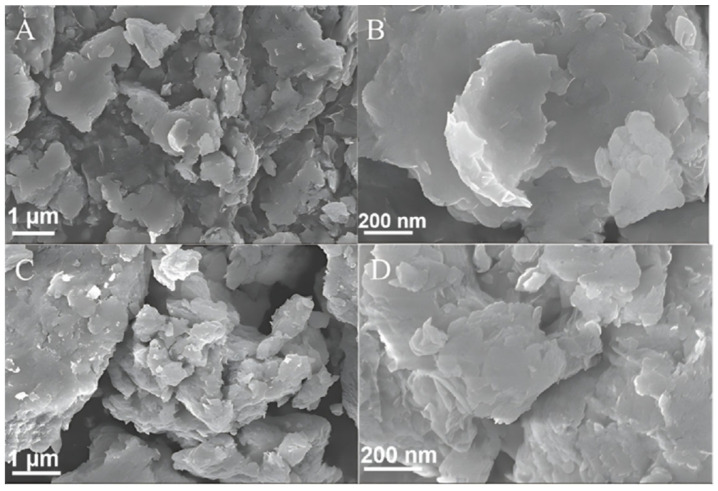
(**A**,**B**) SEM images of ZnIn_2_S_4_ and (**C**,**D**) SEM images of 10% nano g-C_3_N_4_/ZnIn_2_S_4_.

Figure 2 and Figure 3 show the SEM and TEM images of the g-C_3_N_4_ and nano g-C_3_N_4_ samples, respectively, and Figure 4 shows the SEM and TEM images of the ZnIn_2_S_4_ and 10% g-C_3_N_4_/ZnIn_2_S_4_ nano-heterojunctions. In Figure 2 and Figure 3, it can be seen that the g-C_3_N_4_ that we prepared had an irregular layered structure, while nano g-C_3_N_4_ was a lamellar thin nanosheet. Figure 4 shows that the ZnIn_2_S_4_ sample material had a microspherical pleated structure, while the micrograph of the 10% g-C_3_N_4_/ZnIn_2_S_4_ nanohybrid junction material obtained after their composite shows that the lamellar g-C_3_N_4_ grew tightly on the microspherical pleated layers of the ZnIn_2_S_4_. This kind of structure, with obvious lattice stripes, can shorten the diffusion distance of the light-generated carriers, which greatly improves its ability to capture photons, thus improving the photocatalytic performance to a certain extent.

### 2.3. Fourier Transform Infrared Spectroscopy (FTIR) Analysis

**Figure 5:**
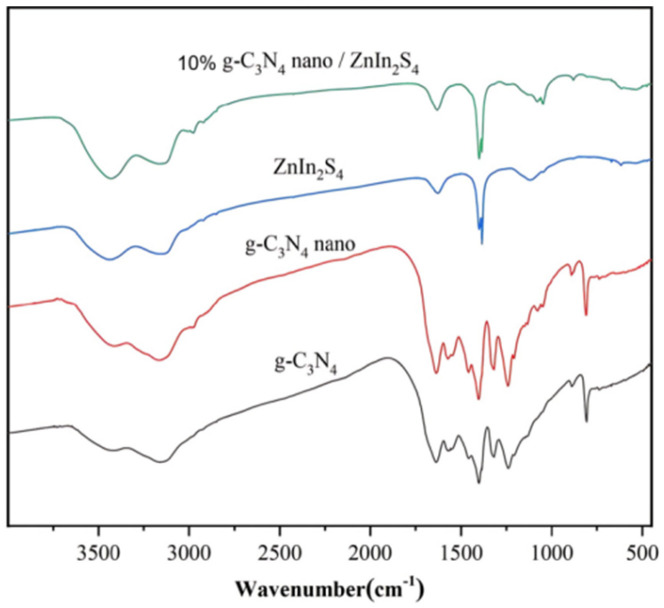
FTIR spectra of g-C_3_N_4_, nano g-C_3_N_4_, ZnIn_2_S_4_, and 10% nano g-C_3_N_4_/ZnIn_2_S_4_.

Figure 5 shows the infrared spectra of the catalysts prepared in this experiment, in which it can be seen that there was a clear absorption band at 2900–3500 cm^−1^ for g-C_3_N_4_, which was caused by the hydroxyl group of H_2_O and N-H in the layered structure of g-C_3_N_4_. There was also a characteristic peak at 810 cm^−1^, which was due to the stretching and vibration of the molecules of the triazinane cyclic analogs. The absorption band at 1130–1240 cm was caused by hydroxyl group absorption in the structure of the sample, and the characteristic peak at 1250–1650 cm was due to the skeleton vibration of the aromatic heterocyclic compounds. The characteristic peaks at 1300–1600 cm^−1^ were due to the adsorption of water molecules by the hydroxyl groups on the surface of the samples. The absorption peaks at g-C_3_N_4_ were all in the nano-heterojunctions of the g-C_3_N_4_/ZnIn_2_S_4_. According to the spectra, the composite 10% nano g-C_3_N_4_/ZnIn S_2_ nano-heterojunction contained the telescopic vibration modes of both the ZnIn_2_S_4_ and g-C_3_N_4_ samples, which indicates that the nano-heterojunction prepared in this experiment contained the presence of the components of ZnIn_2_S_4_ and g-C_3_N_4_. This further verifies that the composite photocatalytic material of the 10% nano g-C_3_N_4_/ZnIn_2_S_4_ nano-heterojunction was successfully prepared.

### 2.4. Photoluminescence (PL) Analysis

**Figure 6:**
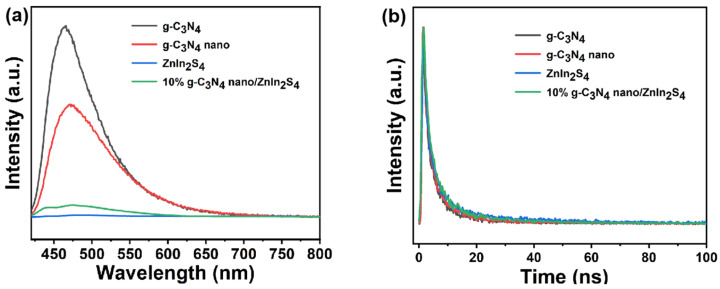
(**a**) Steady-state photoluminescence spectra of different samples. (**b**) Time-resolved photoluminescence decay spectra of g-C_3_N_4_, nano g-C_3_N_4_, ZnIn_2_S_4_, and 10% nano g-C_3_N_4_/ZnIn_2_S_4_.

Photoluminescence (PL) and time-resolved fluorescence decay properties are important tools used to explore the rate of charge generation, separation, and complexation, as the intensity of fluorescence is closely related to the complexation of electrons and holes. Figure 6a compares the fluorescence intensities of g-C_3_N_4_, nano g-C_3_N_4_, Znln_2_S_4_, and 10% nano g-C_3_N_4_/Znln_2_S_4_. It can be clearly seen that Znln_2_S_4_ has almost no obvious absorption peak at the excitation wavelength of 375 nm due to its optical nature. The emission peaks of the 10% nano g-C_3_N_4_/Znln_2_S_4_ composites are relatively weak, which is most likely due to the effective charge transfer between 10% nano g-C_3_N_4_ and Znln_2_S_4_.

The time-resolved fluorescence decay spectra also provide a strong basis for evaluating the separation efficiency of photogenerated charge carriers. Figure 6b shows the time-resolved fluorescence decay curves of the samples evaluated using the double-exponential fitting equation as follows: I(t) = A_1_ × exp($\frac{-t}{\tau_{1}}$) + A_2_ × exp($\frac{-t}{\tau_{2}}$). The average lifetime is $\tau_{ave}=\frac{A_{1}\tau_{1}+A_{2}\tau_{2}}{A_{1}+A_{2}}$. In the figure, it can be seen that the fluorescence lifetime of the pure g-C_3_N_4_ was relatively short, only 7.2 ns. In contrast, the fluorescence average lifetime of 10% nano g-C_3_N_4_/Znln_2_S_4_ reached 8.9 nanoseconds, which is nearly 16% longer than the 7.5 nanoseconds of Znln_2_S_4_. The increase in the average lifetimes of the composite photocatalysts Znln_2_S_4_ and 10% nano g-C_3_N_4_/Znln_2_S_4_ compared to g-C_3_N_4_ suggests that the 2D/2D VDW heterojunction effectively created more high-speed charge transfer paths, which promoted the efficient transfer of photogenerated charges and consequently enhanced the photocatalytic hydrogen production activity.

### 2.5. X-ray Photoelectron Spectroscopy (XPS) Analysis

**Figure 7:**
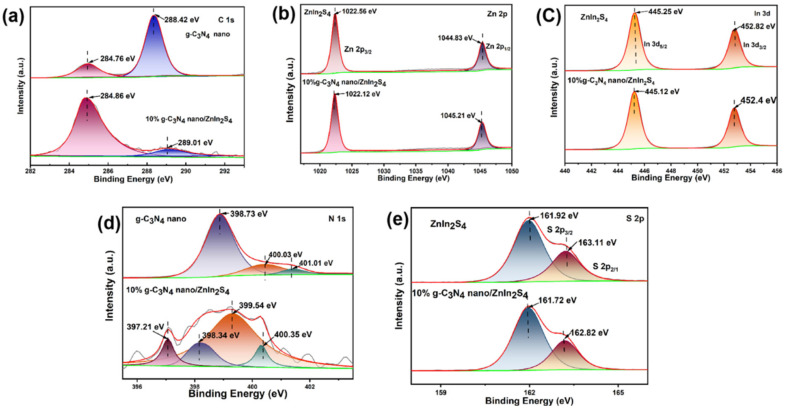
(**a**) High-resolution XPS spectra of C 1s of g-C_3_N_4_ and 10% nano g-C_3_N_4_/Znln_2_S_4_, (**b**) high-resolution XPS spectra of Zn 2p of Znln_2_S_4_ and 10% nano g-C_3_N_4_/Znln_2_S_4_, (**c**) high-resolution XPS spectra of In 3d of Znln_2_S_4_ and 10% nano g-C_3_N_4_/Znln_2_S_4_, (**d**) high-resolution XPS spectra of N 1s of g-C_3_N_4_ and 10% nano g-C_3_N_4_/Znln_2_S_4_, (**e**) high-resolution XPS spectra of S 2p of Znln_2_S_4_ and 10% nano g-C_3_N_4_/Znln_2_S_4_.

XPS further confirmed the composition of the 10% nano g-C_3_N_4_/Znln_2_S_4_ sample. Figure 7a shows that the C 1s spectrum of nano g-C_3_N_4_ has two characteristic peaks located at 288.42 and 284.76 eV. These peaks correspond to the sp^2^-hybridized carbon-containing defects (N-C=N) and graphitic carbon in the carbon-containing aromatic ring of the sp^2^ bonds (C-C, C=C). Typically, the C 1s peak at 284.76 eV originates from carbon-based impurities, which are generated during high-temperature treatment [27,28,29]. This impurity peak increases in intensity as the amount of cyanuric acid increases [30]. Figure 7b shows that the Zn 2p XPS spectrum of Znln_2_S_4_ has two significant peaks located at 1022.56 and 1044.83 eV, which correspond to Zn 2p_3/2_ and Zn 2p_1/2_, respectively, while the 10% Zn 2p_1/2_ peaks are located at 1022.56 and 1044.83 eV, respectively. Meanwhile, the Zn 2p characteristic peaks corresponding to nano 10% g-C_3_N_4_/Znln_2_S_4_ show a slight shift to 1022.12 and 1045.21 eV, respectively. Similarly, Figure 7c shows that the In 3d spectra of Znln_2_S_4_ has two characteristic peaks located at 445.25 and 452.82 eV, which correspond to In 3d_5/2_ and In 3d_3/2_. Similar to the above, the positions of the In 3d peaks corresponding to 10% nano g-C_3_N_4_/Znln_2_S_4_ show a small decrease, shifting to 445.12 and 452.4 eV, respectively. In addition, the N 1s spectrum of nano g-C_3_N_4_ shown in Figure 7d reveals three characteristic peaks at 401.01, 400.03, and 398.73 eV, which correspond to the amino group (C-N-H), the tertiary nitrogen N-(C)_3_ group, and the sp^2^-bonded N (C-N=C), respectively [31,32]. Notably, both the N 1s and C 1s characteristic signals of nano g-C_3_N_4_ were slightly shifted to higher binding energies after forming a heterojunction with the ZnIn_2_S_4_ nanosheets, which could be attributed to the transfer of some of the electrons from the N and C to the S of ZnIn_2_S_4._ In addition, the observed peak at 397.21 eV in the N 1s spectrum of 10% nano g-C_3_N_4_/Znln_2_S_4_ may be related to the N-S bonding, which further proves the existence of a strong interfacial interaction between nano g-C_3_N_4_ and ZnIn_2_S_4_ [33]. Figure 7e shows that the binding energies of S 2p_3/2_ and S 2p_1/2_ are located at 161.92 and 163.11 eV, respectively, which are related to S^2−^ in Znln_2_S_4_ [34]_._ Similarly, the binding energies at 161.72 and 162.82 eV of 10% nano g-C_3_N_4_/Znln_2_S_4_ are lower than those of Znln_2_S_4_. Both the binding energy and the peak intensities of S 2p in 10% nano g-C_3_N_4_/Znln_2_S_4_ become smaller, and, theoretically, the increase in the binding energy is attributed to the decrease in the electron density, resulting in a weakening of the electron shielding effect. On the contrary, the electron density increase leads to a decrease in the binding energy, which is due to the enhancement of the electron shielding effect [35].

### 2.6. UV–Visible Diffuse Reflectance Spectroscopy

**Figure 8:**
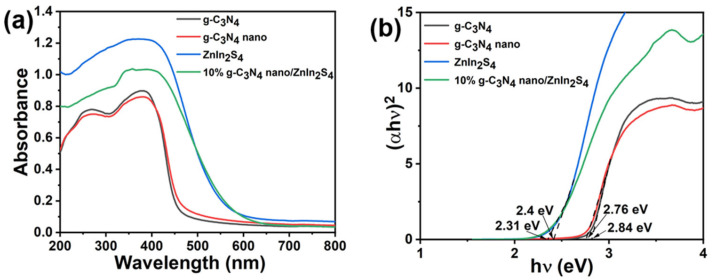
(**a**) UV–Vis diffuse reflectance spectra of g-C_3_N_4_, nano g-C_3_N_4_, Znln_2_S_4_, and 10% nano g-C_3_N_4_/Znln_2_S_4_; (**b**) band gaps of g-C_3_N_4_, nano g-C_3_N_4_, Znln_2_S_4_, and 10% nano g-C_3_N_4_/Znln_2_S_4_.

Figure 8a shows the UV–Vis diffuse reflectance spectroscopy of the different samples. The pristine Znln_2_S_4_ had a broad visible absorption range, with an absorption boundary at about 480 nm, whereas the pure g-C_3_N_4_ exhibited an absorption range from UV to visible, with a steep absorption boundary at about 400 nm. Significant red-shifting of the absorption boundaries of the Znln_2_S_4_ and 10% nano g-C_3_N_4_/Znln_2_S_4_ composites occurred compared to pure g-C_3_N_4_, implying that these complexes trapped visible light more efficiently. Figure 8b shows the band gaps of the different samples. We calculated the band gap values of the materials by applying the Kubelka–Munk method (the specific formula is (αhν)^2^ = K(hν − Eg), where hν represents the photon energy, K is a constant, α is the absorption coefficient, and Eg represents the bandgap energy). By observing the Tauc curves, we obtained the bandgap energies of g-C_3_N_4_, nano g-C_3_N_4_, Znln_2_S_4_, and 10% nano g-C_3_N_4_/Znln_2_S_4_, which were about 2.84, 2.76, 2.4, and 2.31 eV, respectively.

### 2.7. N_2_ Adsorption–Desorption Isotherm

**Figure 9:**
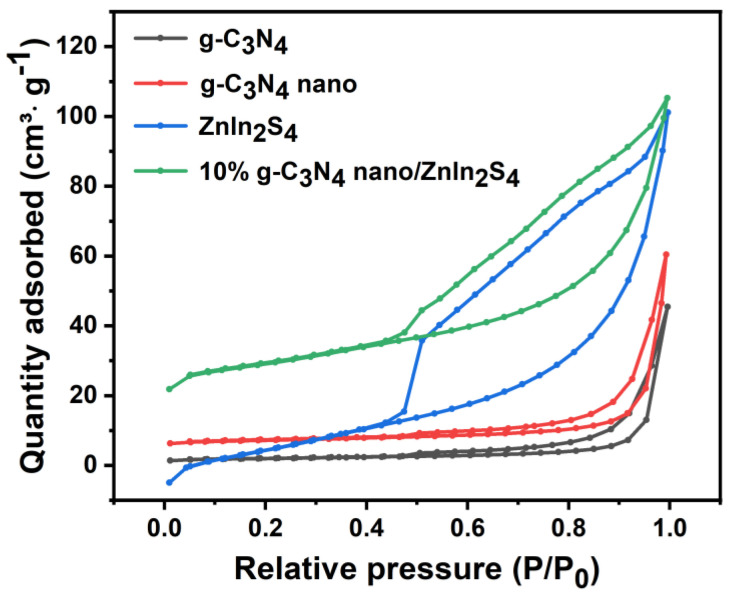
N_2_ adsorption−desorption isotherms of g-C_3_N_4_, nano g-C_3_N_4_, ZnIn_2_S_4_, and 10% nano g-C_3_N_4_/ZnIn_2_S_4_ samples.

Using N_2_ adsorption–desorption isotherms, the specific surface areas and pore size distributions of the g-C_3_N_4_, nano g-C_3_N_4_, ZnIn_2_S_4_, and 10% nano g-C_3_N_4_/ZnIn_2_S_4_ composites were studied. Table 1 shows that the average pore sizes of these materials were 20.05 nm, 19.1 nm, 10.56 nm, and 9.79 nm, respectively, indicating that the ZnIn_2_S_4_ and the composite had smaller pore sizes, and the average pore size was reduced due to the increase in mesopores. In terms of the specific surface area, 10% nano g-C_3_N_4_/ZnIn_2_S_4_ exhibited the largest specific surface area of 90.57 m^2^/g, which is beneficial for increasing the contact area with pollutants and promoting photocatalytic reactions. Additionally, the pore volumes of these materials were 0.07 m^3^/g, 0.09 m^3^/g, 0.18 m^3^/g, and 0.24 m^3^/g, respectively. An increase in the pore volume helps to enhance the adsorption capacity of the catalyst, thereby accelerating the catalytic degradation of pollutants. Figure 9 presents the adsorption–desorption isotherms of g-C_3_N_4_, nano g-C_3_N_4_, ZnIn_2_S_4_, and 10% nano g-C_3_N_4_/ZnIn_2_S_4_. All samples exhibited characteristic IV adsorption isotherms with H3 hysteresis loops, indicating the presence of mesoporous structures.

### 2.8. Photocatalytic Activity Studies

**Figure 10:**
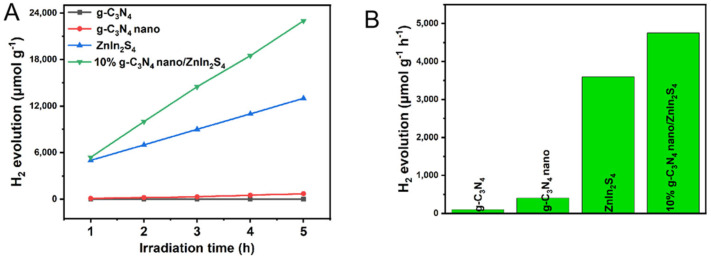
(**A**) Photocatalytic hydrogen production of different samples under visible light for 5 h. (**B**) Hydrogen production rates of different samples.

Figure 10 shows the photocatalytic hydrogen production of the different samples under visible light illumination for 5 h, as well as the rate of hydrogen production. It can be seen that the hydrogen production of both the g-C_3_N_4_ and the g-C_3_N_4_ nanosheets was low and negligible compared to that of the ZnIn_2_S_4_ and 10% nano g-C_3_N_4_/ZnIn_2_S_4_. This was due to the high recombination rate of the photogenerated carrier in the graphene-like layered structure of g-C_3_N_4_. The 10% nano g g-C_3_N_4_/ZnIn_2_S_4_ sample material, obtained by loading 10% g-C_3_N_4_ nanoflakes onto the ZnIn_2_S_4_ semiconductor material, possessed a relatively high hydrogen yield. In Figure 6B, it can be seen that the hy-drogen yield of g-C_3_N_4_ was 32.7372 μmol g^−1^ h^−1^, whereas that of the 10% g-C_3_N_4_/ZnIn_2_S_4_ nanohybrid junction photocatalytic material was as high as 4772.6404 μmol g^−1^ h^−1^, which is equivalent to 100 times that of the g-C_3_N_4_ photocatalytic material. This also means that the 10% g-C_3_N_4_/ZnIn_2_S_4_ nano-heterojunction prepared in this ex-periment possessed high photocatalytic activity. This is due to the fact that the hetero-junction structure formed by the composite of g-C_3_N_4_ and ZnIn_2_S_4_ semiconductors effectively inhibits the recombination of photogenerated carriers, thus improving the photocatalytic performance of the material to a large extent.

### 2.9. Stability Analysis of 10% g-C_3_N_4_/ZnIn_2_S_4_ Nano-Heterojunction

**Figure 11:**
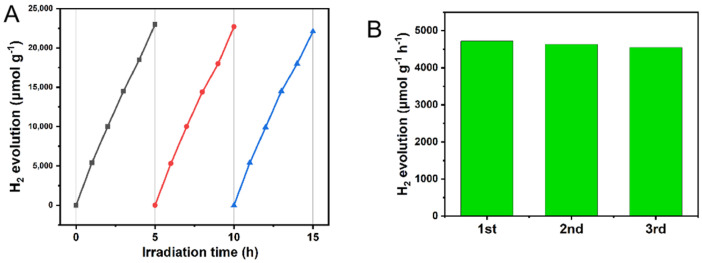
(**A**) Cyclic hydrogen production plot of prepared 10% nano g-C_3_N_4_/ZnIn_2_S_4_. (**B**) Cyclic hydrogen production rate plot.

Based on the above experiments and analyses, it is clear that the 10% g-C_3_N_4_/ZnIn_2_S_4_ nanohybrid junction composites prepared in this experiment can effectively improve the photocatalytic activity. However, in real life and production, developing materials with high catalytic activity is only one of the goals of our research; their practicality is an indicator that we cannot ignore. Therefore, while pursuing high catalytic activity, we should also ensure that the prepared photocatalytic materials have a long life and stability. In this experiment, we also investigated the stability of the 10% g-C_3_N_4_/ZnIn_2_S_4_ nano-heterojunction composite photocatalytic materials for hydrogen production, as shown in Figure 11A,B. After three cycles of experimental testing, with 5 h of irradiation each time, the test results show that the catalytic activity of the sample catalysts did not decrease significantly during the test, which indicates that the 10% g-C_3_N_4_/ZnIn_2_S_4_ nano-heterojunction composite photocatalytic materials prepared in this experiment have a long life and stability. The ZnIn_2_S_4_ nano-heterojunction catalytic material did not decompose under visible light irradiation and still maintained its high catalytic activity, which also strongly verifies that the composites prepared in this experiment have high stability and reusability.

### 2.10. Mechanism Analysis

**Figure 12:**
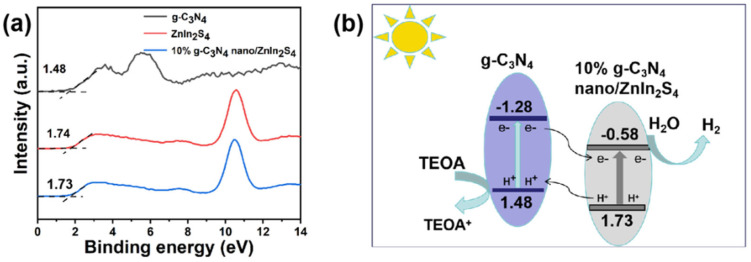
(**a**) Valence band spectra of g-C_3_N_4_, nano g-C_3_N_4_, Znln_2_S_4_, and 10% nano g-C_3_N_4_/Znln_2_S_4_. (**b**) Photocatalytic mechanism diagram of 10% g-C_3_N_4_/Znln_2_S_4_ nano−heterojunction.

The Schottky barrier formed at the metal/semiconductor interface promotes electron–hole separation in photocatalysis, while the metal acts as an electron sink. The photoinduced electrons in noble metals and holes in the VB of semiconductors participate in subsequent redox reactions. The whole process increases the charge carrier lifetime and improves the photocatalytic activity. The unique localized surface plasmon resonance (LSPR) of noble metal NPs can boost photocatalytic activity by increasing the incoming photon absorption. Furthermore, the facile tunability of LSPR (i.e., by adjusting the size and shape) allows for a broad spectrum of light absorption [36]. Conduction band (CB) potentials of −0.58 and −1.28 V (relative to the NHE) were calculated for 10% nano g-C_3_N_4_/Znln_2_S_4_ and g-C_3_N_4_, respectively, based on the equation E_CB_ = E_VB_ − E_g_, according to the bandgap energy data map in Figure 12. The energy band alignment in the figure indicates that the E_CB_ of g-C_3_N_4_ was more negative than that of 10% nano g-C_3_N_4_/Znln_2_S_4_, while the E_VB_ position of 10% nano g-C_3_N_4_/Znln_2_S_4_ was below that of g-C_3_N_4_. Due to the energy matching between 10% nano g-C_3_N_4_/Znln_2_S_4_ and g-C_3_N_4_, the transfer of the photogenerated holes or electrons between the two semiconductors is considered a type II heterostructure mechanism. Under visible light irradiation, 10% nano g-C_3_N_4_/Znln_2_S_4_ and g-C_3_N_4_ were excited and produced electron–hole pairs. The photoinduced electrons were transferred from the CB of g-C_3_N_4_ to the CB of 10% nano g-C_3_N_4_/Znln_2_S_4_ through the tight two-dimensional heterojunction interface, and then H^+^ was reduced to H_2_. Photogenerated holes at the VB energy level of 10% nano g-C_3_N_4_/Znln_2_S_4_ can be spontaneously injected into the VB energy level of g-C_3_N_4_. In addition, due to the two-dimensional structure of the ultrathin g-C_3_N_4_ and 10% nano g-C_3_N_4_/Znln_2_S_4_, the photoinduced carriers are able to migrate rapidly to the surface for efficient subsequent reactions. Compared to ZnIn_2_S_4_ and g-C_3_N_4_ alone, the proper arrangement of the energy band structures in the composites facilitates the separation of photoinduced carriers and thereby promotes photocatalytic H_2_ release.

## 3. Experimental Section

### 3.1. Preparation of Photocatalysts

#### 3.1.1. Preparation of g-C_3_N_4_ Nanoflakes

We prepared g-C_3_N_4_ using a calcination method. The experimental steps were as follows. First, 3.0000 g of melamine was accurately weighed and then ground into a fine powder in a quartz mortar. The ground melamine was then transferred and poured into a ceramic crucible and put into a tube furnace, where it reacted for 4 h under a temperature of 550 °C (the rate of the temperature increase was 5 °C/min). Argon gas was introduced into the tube furnace, and the reaction was completed. After the tube furnace temperature was reduced to room temperature, the sample was taken out. The obtained yellow powder was the g-C_3_N_4_ semiconductor photocatalytic material, of which 0.5000 g was weighed and put into the tube furnace. It reacted for 2 h at a temperature of 550 °C (with a heating rate of 5 °C/min). Argon gas was introduced. At the end of the reaction, and after the temperature of the reaction system was reduced to room temperature, the obtained yellow sample was removed and ground. The light yellow powder obtained was the prepared g-C_3_N_4_ nanoflake photocatalytic material.

#### 3.1.2. Preparation of ZnIn_2_S_4_ Material

We prepared ZnIn_2_S_4_ semiconductor nanomaterials with a hexagonal crystal type using the hydrothermal method; the specific steps were as follows. We weighed 1 mmol (0.2975 g) of zinc nitrate hexahydrate, 2 mmol (0.6017 g) of indium nitrate hydrate, and 6 mmol (0.4508 g) of thioacetamide, and combined them in a beaker. Then, we added 30 mL of water and mixed it well before placing the mixture into a rotor with a magnetic stirrer. The mixed solution was transferred to a reaction kettle, and the sealed reaction kettle was placed in an electrically heated constant-temperature blast-drying oven. The temperature of the reaction system was set to 180 °C, and the reaction was carried out for 12 h. When the reaction was complete, the temperature of the constant-temperature oven was lowered to room temperature. The samples obtained from the reaction were washed with ultrapure water 3 times, and then washed with anhydrous ethanol 3 times. Then, they were placed in a drying oven at 70 °C to dry. The dried samples were ground and then dried again at 70 °C. The obtained yellow powder was the sample of ZnIn_2_S_4_ photocatalytic material.

#### 3.1.3. Preparation of 10% g-C_3_N_4_/ZnIn_2_S_4_ Nano-Heterojunction Composites

We weighed out 0.0149 g of g-C_3_N_4_, 1 mmol (0.2975 g) of zinc nitrate hexahydrate, 2 mmol (0.6017 g) of indium nitrate hydrate, and 6 mmol (0.4508 g) of thioacetamide and placed them in a beaker. Then, we added 30 mL of water, and it was mixed under ultr−sonic conditions for 20 min until a uniform solution was obtained. Then, it was put into the rotor and stirred with a magnetic stirrer for 20 min. The solution was transferred to the reaction kettle, and the sealed reaction kettle was placed in the electric constant-temperature blast-drying oven. The temperature was set to 180 °C, and the reaction time was 12 h. After the reaction was completed and the oven cooled down to room temperature, the samples obtained from the reaction were washed with ultrapure water 3 times and then washed with anhydrous ethanol 3 times, under the conditions of a centrifuge rotation speed of 8000 r/min for 3 min. Then, the samples were put into a drying oven and dried at a temperature of 70 °C. The yellow powder obtained from grinding was the 10% g-C_3_N_4_/ZnIn_2_S_4_ composite photocatalytic material sample.

### 3.2. Material Characterization

The powder X-ray diffraction (XRD) patterns of the as-prepared samples were analyzed using a Bruker D8 Advance X-ray diffractometer with Cu Kα radiation. The Bruker D8 Advance X-ray diffractometer is manufactured by Bruker Company, Germany. Bruker’s headquarters is located in Saarbrücken, Germany. The morphology was observed using a Hitachi S-4800 field emission scanning electron microscope (SEM) and a JEOL JEM 2100F transmission electron microscope (TEM). The Hitachi S-4800 Field Emission Scanning Electron Microscope (SEM) is manufactured by Hitachi Ltd., with its headquarters located in Chiyoda-ku, Tokyo, Japan. The JEOL JEM 2100F Transmission Electron Microscope (TEM) is manufactured by JEOL Ltd., which is also based in Chiyoda-ku, Tokyo, Japan. The X-ray photoelectron spectra (XPS) were recorded using a Thermo ESCALAB 250XI. The manufacturer of the Thermo ESCALAB 250XI equipment is Thermo Fisher Scientific, headquartered in Waltham, MA, USA. N_2_ adsorption–desorption isotherms were recorded at 77 K using a Quantachrome Autosorb-IQ-2C analyzer. The photoluminescence (PL) spectra were measured using a FLS-980 (Edinburgh, UK). The Quantachrome Autosorb-IQ-2C is manufactured by Quantachrome Instruments, with its headquarters located in Boca Raton, FL, USA. The FLS-980 fluorescence spectrometer is manufactured by Edinburgh Instruments, headquartered in Livingston, UK.

### 3.3. Photocatalytic Performance Test

In this experiment, we used a Labsilar-III (AG) photolyzed water hydrogen system, produced by Beijing Porphyry Technology Co. (Beijing, China). This photolyzed water system has a vacuum system and an online system, and the experiment was carried out at atmospheric pressure to produce hydrogen for online sampling and testing to ensure the accuracy and consistency of the experiment. When the semiconductor is irradiated with energy greater than or equal to its forbidden bandwidth, the valence electrons within the semiconductor are excited, jumping to the conduction band, which causes photogenerated electron–hole separation. In this experiment, the photocatalytic hydrogen production test was carried out under the irradiation condition of a xenon lamp filtering out the ultraviolet light instead of sunlight, and the reaction device was in a vacuum state. Triethanolamine was used as the hole sacrificial agent, chloroplatinic acid was the co-catalyst, and the xenon lamp current was 15 mA. When the conduction band potential of the semiconductor material tested is more negative than the hydrogen electrode potential, its valence band potential is more positive than the oxygen electrode potential, and a redox reaction can occur to reduce the water to hydrogen. The experimental feeding time interval was one hour to obtain the amount of hydrogen prepared using photocatalysis for each sample. This cycle was used until the reaction was complete, and the photocatalytic activity of each sample was analyzed based on the test data.

## 4. Conclusions

In this experiment, g-C_3_N_4_/ZnIn_2_S_4_ nano-heterojunction composites were prepared using a combination of ultrasonication and hydrothermal methods, and g-C_3_N_4,_ prepared using the calcination method, was loaded onto ZnIn_2_S_4_ catalytic material. Then, the composites were characterized using XRD, SEM, UV, and FTIR, and the compositions of the composites were verified, i.e., g-C_3_N_4_/ZnIn_2_S_4_ nano-heterojunction composites were successfully prepared. Under visible light irradiation, the composite material showed higher photocatalytic activity compared to pure g-C_3_N_4_ and ZnIn_2_S_4_. This was due to the heterojunction structure formed by the interfacial contact of the two semiconducting materials, g-C_3_N_4_ and ZnIn_2_S_4_, which effectively inhibited the recombination of the photogenerated carriers and improved the separation efficiency of the photogenerated electron–hole pairs, thus increasing the catalytic activity. At the same time, the heterojunction structure made the composite material more efficient and stable.

## Figures and Tables

**Table 1:** Pore structure parameters of g-C_3_N_4_, nano g-C_3_N_4_, ZnIn_2_S_4_, and 10% nano g-C_3_N_4_/ZnIn_2_S_4_ samples.

Sample	S_BET_ (m^2^·g^−1^)	V_Total_ (m^3^·g^−1^)	D_Average_ (nm)
g-C_3_N_4_	6.9	0.07	20.05
g-C_3_N_4_ nano	9.36	0.09	19.1
Znln_2_S_4_	65.85	0.18	10.56
10% g-C_3_N_4_ nano/Znln_2_S_4_	90.57	0.24	9.79

## Data Availability

All data presented are available in this article.
